# Extent of κ‐casein hydrolysis during renneting of bovine milk: A critical assessment of the analytical and estimation approaches

**DOI:** 10.1002/fsn3.3868

**Published:** 2023-12-12

**Authors:** Joseph F. Kayihura

**Affiliations:** ^1^ Advanced Food Systems Research Unit, Institute for Sustainable Industries and Liveable Cities, College of Health and Biomedicine Victoria University Melbourne Victoria Australia

**Keywords:** bovine milk, caseinoglycomacropeptide, caseinomacropeptide, cheese, rennet, κ‐CN hydrolysis

## Abstract

Renneting is an enzymatic process that turns milk into curd which is then transformed into cheese. Rennet‐induced coagulation of caseins (CNs) is the critical step during this process and the key is the primary hydrolysis of κ‐CN's Phe_105_‐Met_106_ bond by chymosin. This article comprehensively reviews the existing data on the extent/degree of κ‐CN hydrolysis during renneting of bovine milk and critically evaluates its determination methods. The data show that under normal cheese‐making conditions, milk gelation occurs at a degree of κ‐CN hydrolysis <80%, which varies due to several factors including analytical and estimation approaches. The common approach involves isolating the macropeptides released, by precipitating whey proteins and residual CN in 1%–12% trichloroacetic acid (TCA), then assuming that the maximum amount obtained is 100% κ‐CN hydrolysis. The drawback is that the estimated degree of κ‐CN hydrolysis may be higher than the actual value as TCA partially precipitates the macropeptide fractions. Moreover, macropeptide isolation seems unnecessary based on current advances in chromatographic and electrophoretic techniques. The present work proposes a simple mass balance‐based approach that will provide accurate estimates in future studies. The accuracy of measuring the degree of κ‐CN hydrolysis has implications on the precision of the data in relation to its partitioning (% distribution between the curd and whey) which is essential for improving whey quality.

## Graphical abstract

- κ‐casein hydrolysis during renneting—a critical step for cheese‐making.
- The degree of κ‐casein hydrolysis by rennet has implications on its partitioning and whey quality.
- Influential factors include analytical and estimation approaches.

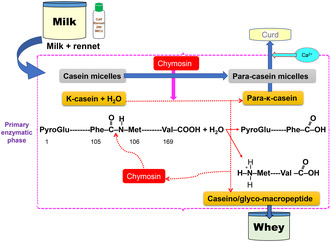

## 1 INTRODUCTION

Rennet‐induced coagulation of CNs (approximately 80% of bovine milk proteins) is the critical step during the processing of many cheese varieties. The key to achieving the coagulation of CNs is the primary proteolytic/hydrolytic action of rennet on κ‐CN. CNs (41% α_s1_, 8% α_s2_, 37% β, and 14% κ) (Kayihura, 2023c) in milk occur primarily in the form of a stable colloidal dispersion owing to κ‐CN's functional role as a (i) chain terminator during micellar assembly due to its lack of phosphoserine clusters that limit further networking and (ii) micellar stabilizer (Huppertz, 2013; Lucey, 2022). κ‐CN located mainly on the surface as a hairy layer (or brush) provides micellar stability by steric and electrostatic repulsive interactions between its hydrophilic C‐terminal moieties (Mackinlay & Wake, 1971; Vreeman et al., 1986). The layer was estimated to be 4.5–7 nm thick (Holt & Horne, 1996; van Hooydonk, Hagedoorn, & Boerrigter, 1986) although 10–12 nm has also been reported (Dalgleish, 1998; Sandra et al., 2007).

The discovery of κ‐CN as a stabilizer or protective colloid dates back to 1950s (Waugh & von Hippel, 1956) whereas the hairy layer model was proposed in 1996 but later supported by experiments in 1981 (Holt & Horne, 1996; Walstra et al., 1981). Thus, a suitable explanation for the rennet‐induced destabilization of CN micelles resulting in milk‐curd formation is that rennet enzymes cut off the stabilizing hairy layer allowing the hydrophobic and Ca‐sensitive core components to aggregate (Horne & Lucey, 2017; Huppertz et al., 2018). Chymosin, the main enzyme in rennet, specifically hydrolyzes κ‐CN's Phe_105_‐Met_106_ bond producing two peptides, namely, the hydrophobic N‐terminal fraction (f1‐105) known as para‐κ‐CN and the hydrophilic C‐terminal fraction (f106‐169) referred to as caseinomacropeptide (CMP) also called aglycosylated caseinomacropeptide (aCMP) if no carbohydrate, or glycomacropeptide (GMP) also called caseinoglycomacropeptide (cGMP) or glycosylated caseinomacropeptide (gCMP) if one or more carbohydrates are attached (Boutrou et al., 2008; Karimidastjerd & Gulsunoglu‐Konuskan, 2023; Lucey, 2022; Sunds et al., 2019). The primary structures of κ‐CN and both of its peptides have been established (Mercier et al., 1973) and proportions of glycosylated forms may vary depending on genetic factors (Bonfatti et al., 2014).

It is believed that there is a minimum degree of κ‐CN hydrolysis necessary to induce milk gelation. Therefore, the primary enzymatic phase and subsequent aggregation of the para‐CN micelles also referred to as the secondary phase are fundamental to cheese‐making (Kalan & Woychik, 1965; Kelly et al., 2008; Lucey, 2022). However, quantitative aspects require careful assessment as there are marked variations in renneting conditions some of which are irrelevant to cheese‐making, and most methods available lead to partial estimates. This review aims to comprehensively analyze the existing data on the primary phase of rennet action on κ‐CN, underline the major factors, and provide a critical assessment of the analytical and estimation approaches. Specifically, the article attempts to answer the following questions: (a) what is the proportion (%) of the C‐terminal macropeptide (CMP and GMP) fraction of κ‐CN? (b) what is the distribution (%) of GMP? (c) what is the extent of hydrolysis (%) necessary to induce milk gelation under normal cheese‐making conditions? (d) what is the most reliable method to determine the degree of κ‐CN hydrolysis during renneting? The criteria for the articles selected (Tables 1 and 2) were as follows: (1) original articles, (2) sample type: bovine milk, (3) coagulant: calf rennet, chymosin, or recombinant chymosin, and (4) the coagulant strength (IMCU) indicated.

**TABLE 1:** Degree of κ‐CN hydrolysis (%) during renneting, stage or time of measurement, type of milk sample, amount of rennet (International milk clotting units, IMCU/mL of milk) as well as type and strength of rennet used.

Degree (%)	Stage and/or time (min)	Sample type	Rennet (IMCU/mL)	Rennet type and strength	References
60	Gelation	Nonfat dry milk, 12% TS	0.00768	>95% chymosin	He (1990)
~68	Gelation	Nonfat dry milk, 36% TS	0.00768		
<60	Gelation	Above samples with 0.01–0.80 M CaCl_2_	0.00768		
<35	Gelation	Nonfat dry milk, 12% TS with 0.05 M CaCl_2_	0.00768		
85	Gelation (4 h)	Fresh skim milk	0.0075	Rennet, 150 IMCU/mL	Vasbinder et al. (2003)
85	Gelation (4 h)	Heated skim milk (70°C/10 min)	0.0075		
67–95	After 4 h	Heated skim milk (90°C/10 min)	0.0075		
75	Gelation at pH 5.8	Reconstituted skim milk, 10% TS	0.09975	Chymostar, 570 IMCU/mL	Li and Dalgleish (2006)
55	Gelation at pH 5.4	Same sample above	0.01653		
100	After 1 h	Commercial pasteurized skim milk (3.4% protein)	0.18	Fermentation produced chymosin, 180 IMCU/mL	Bansal et al. (2007)
50	Coagulation (~15 min)	Skim milk with 2.75% CN, pH 5.8	0.01	Chy‐Max Extra, strength not shown	Karlsson et al. (2007)
<20	Coagulation (27 min)	Ultra‐filtered (UF) skim milk with 19.8% CN, pH 5.8	0.01		
60–67	After 7 h	Reconstituted skim milk (10%)	0.0036	Recombinant chymosin, 180 IMCU/mL	Renan et al. (2007)
50	After 7 h	Reconstituted skim milk (10%) and heated (90°C/10 min)	0.0036		
65–70	Flocculation	Skim milk	0.018–0.07	Chymostar, double strength	Sandra et al. (2007)
>90	Gelation (45–143 min)	Skim milk	0.018–0.07		
100	Formation of stiff gel	Raw milk	0.058	Chymogen, 290 IMCU/mL	Taylor and Woonton (2009)
32.7–85.4	Formation of stiff gel depending on heat (1–3 h)	Raw milk heated at: 65°C/15 s; 72°C/15 s; 90°C/15 s; 100°C/10 min	0.058		
11	Gelation, pH 5.4	Preheated (85°C/300 s) skim milk	0.000314	Chymostar, strength not shown	Cooper et al. (2010)
26	Gelation, pH 5.3	Preheated skim milk (85°C/20 s)	0.00126		
80, 95, 100	Flocculation, gelation (39.3 min), ~40 min	Fresh skim milk	0.018	Chymostar, single strength rennet	Titapiccolo, Corredig, and Alexander (2010)
80, 95, 100	Flocculation, gelation (37.5 min), final	Skim milk	0.018	Chymostar, single strength rennet	Titapiccolo, Alexander, and Corredig (2010)
75, 90, 100	Flocculation, gelation (25.6 min), final	Homogenized milk (34.5 MPa)	0.018		
78–82	Gelation (109 min)	Pasteurized skim milk (74°C/23 s) & 2, 3× UF concentrates	0.00296	Chy‐max Ultra, 790 IMCU/mL	Salvatore et al. (2011)
90	Gelation (40–50 min)	Skim milk (2.9% protein) and 3, 5× UF concentrates	0.034	Chy‐max Ultra, 790 IMCU/mL	Sandra et al. (2011)
>90	Gelation point (30.3 min)	Fresh skim milk	0.018	Chymostar, single strength	Gaygadzhiev et al. (2012)
94–98	Gelation (53–54 min)	Skim milk	0.034	Chy‐Max Ultra, 790 IMCU/mL	Sandra et al. (2012)
85–90	Gelation (32–39 min)	Skim milk with 1 mM CaCl_2_	0.034		
100	~60 min	Skim milk without or with 1 mM CaCl_2_	0.034		
80–85	40–45 min	Milk protein concentrate (MPC) in water, 6% protein	0.034	Chy‐max Ultra, 790 IMCU/mL	Sandra and Corredig (2013)
40, 85	Gelation (10.2 min), >60 min	MPC in water, 6% protein, with 0.04% CaCl_2_	0.034		
>85	Gelation (38.6 min)	MPC in skim milk, 6% protein	0.034		
85	Gelation (51.5 min)	MPC in water (6% protein), dialyzed against skim milk	0.034		
<70	Gelation (21 min)	Commercial pasteurized skim milk (3.2% protein), pH 6	0.034	Chy‐max Ultra, 790 IMCU/mL	Eshpari et al. (2015)
85	After ~70 min	Commercial pasteurized skim milk (3.2% protein), pH 6	0.034		
>90	After 40 min	Individual Jersey and Holstein skim milk with different genetic variants of κ‐CN	0.035	Chy‐max Extra, 615 IMCU/mL	Jensen et al. (2015)
10, 20, 25	15 min	Commercial pasteurized skim milk, pH 6.62	0.01, 0.02, 0.03	Chy‐Max Plus, 200 IMCU/mL	Sinaga et al. (2016)
50	30 min	Same sample above	0.03		
>70	After 60 min	Same sample above	0.03		
100	After 90 min	Same sample above	0.03		
60	Aggregation point	Same sample above	0.03		
85	Gelation (59–60 min)	Commercial skim milk (3.29% protein, pH 6.66) and 3, 5× UF concentrates	0.031	Chy‐max Ultra, 790 IMCU/mL	Zhao and Corredig (2016)
85–90	Gelation (80–100 min)	Same samples above with 300 mM NaCl	0.031		
70	Gelation (>30 min)	CN solution (2.64%, pH 6.6–6.8) with whey protein ratio of 4:1	0.035	Chy‐max Plus FPC, 200 IMCU/mL	Gamlath et al. (2018)
70	Gelation (>30 min)	Same sample above with whey protein ratio of 4:1	0.07		
50	Gelation (<14 min)	Same sample above with whey protein ratio of 0.03:1	0.035		
67	40 min	Fresh skim milk, pH 6.5	0.09 IMCU/mL	Chymax Plus, 200 IMCU/mL	Nilsson et al. (2020)
164	40 min	Fresh skim milk, pH 6.5	0.09 IMCU/mL		
52	40 min	Fresh skim milk, pH 6.5	0.09 IMCU/mL		

**TABLE 2:** Isolation and test methods applied to quantify the extent of κ‐CN hydrolysis by rennet and the results presented as an indication of the calculation/estimation approach.

Isolation method	Test method	Results presented/reported	References
8% TCA	Beckman high‐performance analyzer, System 6300 & spectrophotometric	Moles of amino acids/mL and % hydrolysis at clotting, but calculations are not clearly shown	He (1990)
2% Acetic acid/Na‐acetate	RP‐HPLC	Degree of hydrolysis (CMP, % of values obtained for fresh milk)	Vasbinder et al. (2003)
2, 8 & 12% TCA	RP‐HPLC	CMP (% of values obtained for fresh milk)	
	RP‐HPLC	Degree of hydrolysis (% of values obtained for fresh milk)	
8% TCA	RP‐HPLC	GMP (% of maximum value for unheated milk)	Li and Dalgleish (2006)
8% TCA	RP‐HPLC	Peak area vs. time (maximum peak considered 100%)	Bansal et al. (2007)
12% TCA	RP‐HPLC	Storage modulus vs. hydrolysis of κ‐CN (%), calculation not shown	Karlsson et al. (2007)
pH 4.6	RP‐HPLC	CMP (% of values obtained using unheated milk for 24 h)	Renan et al. (2007)
8% TCA	RP‐HPLC	CMP (% calculated using the fit of the first‐order reaction)	Sandra et al. (2007)
Cation‐exchange	RP‐HPLC	GMP (% of GMP released from raw milk)	Taylor and Woonton (2009)
2% TCA	RP‐HPLC	CMP (% of maximum peak area for unheated milk with 0.0351 IMCU/mL rennet)	Cooper et al. (2010)
2% TCA	RP‐HPLC	CMP (% of the maximum assumed to be 100%)	Titapiccolo, Corredig, and Alexander (2010)
2% TCA	RP‐HPLC	CMP (% of maximum area of control taken as 100%)	Titapiccolo, Alexander, and Corredig (2010)
2% TCA	RP‐HPLC	CMP peak area (% of maximum peak area produced with 0.0710 IMCU/mL)	Salvatore et al. (2011)
2% TCA	RP‐HPLC	CMP (% of maximum peak area considered 100%)	Sandra et al. (2011)
2% TCA	RP‐HPLC	CMP (% relative to the maximum peak area for each sample)	Gaygadzhiev et al. (2012)
2% TCA	Referred to Chaplin and Green (1980)	CMP (% of maximum peak area for each sample considered 100%)	Sandra et al. (2012)
2% TCA	RP‐HPLC	CMP (% of maximum peak area for each sample considered 100%)	Sandra and Corredig (2013)
2% PCA	RP‐HPLC	CMP (% of maximum peak area for each sample considered 100%)	Eshpari et al. (2015)
1% TCA	LC–MS	Reported hydrolyzed κ‐CN (% of intact κ‐CN) but actual results presented are extracted ion chromatographic area vs. time	Jensen et al. (2015)
6% TCA	RP‐HPLC	CMP (% of maximum peak area considered as 100%)	Sinaga et al. (2016)
2% TCA	RP‐HPLC	CMP (% of maximum peak area considered as 100%)	Zhao and Corredig (2016)
4.1% TCA	RP‐HPLC	CMP concentration (normalized to the maximum)	Gamlath et al. (2018)
	CE	ratios of para‐κ‐CN, CMP and intact κ‐CN to initial κ‐CN	Nilsson et al. (2020)

## 2 PROPORTION OF TOTAL MACROPEPTIDE FRACTION OF BOVINE κ‐CN

κ‐CN in bovine milk is 12%–15% of total CN or 3–4 g/L of milk (Donnelly & Barry, 1983; Léonil & Mollé, 1991; Lucey, 2022; Nilsson et al., 2020; Phelan, 1981; Wake, 1959; Waugh & von Hippel, 1956) and the macropeptide in cheese whey is 1.2–1.68 g/L (Doultani et al., 2003; Manso & López‐Fandiño, 2004). Therefore, if the actual cheese yield (Cheddar and Gouda, as examples) is 10%–11% of milk, the total macropeptide would be approximately 35.6%–37.8% of κ‐CN. This is in line with 36.4%–37.9% based on the monomeric molecular weights of 6.8 KDa for CMP and ~8 KDa for GMP (Reddy & Kinsella, 1990; Shin & Jang, 2002; Swaisgood, 1975) and 19–22 KDa for nonglycosylated and glycosylated κ‐CN (Huppertz, 2013; Swaisgood, 1975; Swaisgood et al., 1964), considering up to five trisaccharide units each contributing 0.657 KDa (Swaisgood, 1975). Both ranges are close to theoretical values of 4% of total CN or 1/3 of κ‐CN equivalent to 33% (Chapman, 1981), 30% of total κ‐CN nitrogen (Beeby & Nitschmann, 1963) as well as the 4%–5% of total CN nitrogen (Alais et al., 1953) equivalent to a maximum of 33.3% based on κ‐CN content of 15%. This suggests that the degree of hydrolysis reported in terms of macropeptide (CMP and GMP) release (% of maximum) must demonstrate that the maximum was estimated based on initial κ‐CN content and was approximately 36%–38% of total κ‐CN. However, although values would vary depending on the genetic variant and distribution of GMP discussed below, this was not clearly elucidated in all relevant studies found in the literature.

## 3 DISTRIBUTION OF GMP IN BOVINE κ‐CN

According to literature (Caroli et al., 2009), bovine κ‐CN appears classified into 14 genetic variants (A, A^I^, B, B^2^, C, D, E, F^1^, F^2^, G^1^, G^2^, H, I, and J) of which AA and BB are the most common, GG and HH are common while the rest are rather common or rare. Heterozygous variants such as AB, AE, and BE have also been identified (Hallén et al., 2010; Jensen et al., 2015). The AA variant is considered the parent protein with 169 amino acids whereas its caseinomacropeptide fraction consists of 64 amino acids (f106–169) and contains all the posttranslational phosphorylation and glycosylation. The phosphorylation and glycosylation levels vary from 0 to 3P and 0–6 residues, respectively (Huppertz, 2013; Sheng et al., 2022), whereas up to nine glycan residues were identified for BB variant (Vreeman et al., 1986). The five major glycoforms have been reported (Sunds et al., 2019) and all glycans are known to be attached to threonine residues (Huppertz, 2013). The distribution of GMP appears to be important because numerous studies have shown that the rate of GMP cleavage is slower in comparison with the CMP isoform, and this behavior was attributed to higher electronegativity of the former that retards the rennet access to the active site (Ferron‐Baumy et al., 1992; Jensen et al., 2015; Shin & Jang, 2002; van Hooydonk et al., 1984; Wheelock & Knight, 1969). Although some studies agree on 40%–50% with BB variant exhibiting the highest levels (Bonfatti et al., 2014; Thomä et al., 2006), the degree of glycosylation found in literature is not consistent and ranges from <20% (Sheng et al., 2022) to >90% (Vasbinder et al., 2003). It varies with total κ‐CN content and is dependent on animal genetics and isolation techniques; hence, the actual distribution may still be controversial.

## 4 DEGREE OF κ‐CN HYDROLYSIS UNDER CONDITIONS RELEVANT TO CHEESE‐MAKING

**FIGURE 1:**
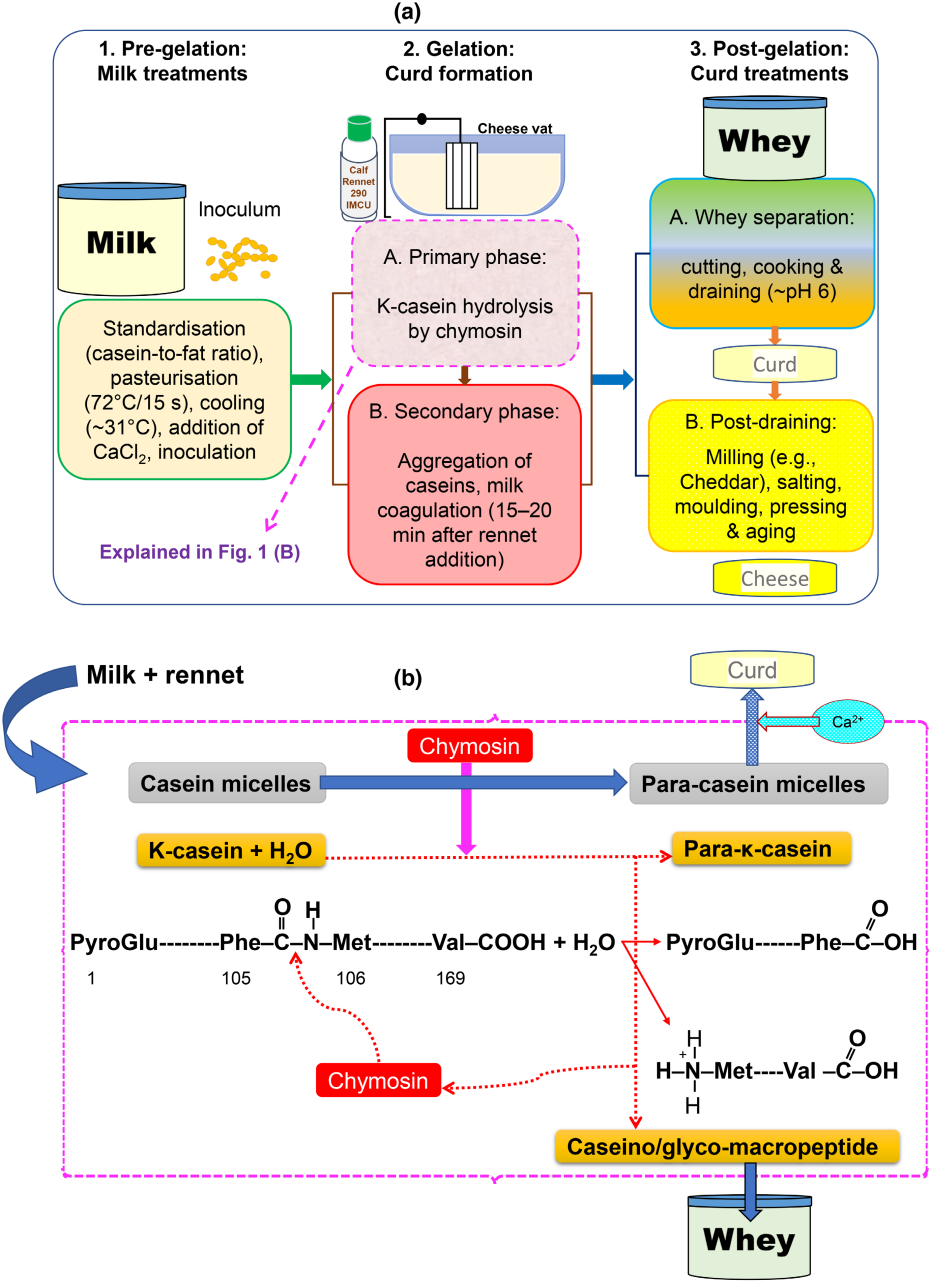
(a): Major stages of the general aged cheese‐making process. (b): Primary enzymatic phase of κ‐CN hydrolysis by chymosin at Phe105‐Met106 bond which releases the caseinomacropeptide [soluble C‐terminal fraction (f106–169)] into whey allowing para‐κ‐CN [hydrophobic fraction (f1–105)] together with other micellar components (collectively referred to as para‐casein micelles) to aggregate. Further details of the reaction mechanism are available (Palmer et al., 2010; Yegin & Dekker, 2013.

Cheese‐making generally involves three major stages (Figure 1a): (1) milk pretreatments including standardization (e.g., casein‐to‐fat ratio), pasteurization (e.g., 72°C/15 s), cooling to renneting temperature (e.g., 30–32°C), addition of CaCl_2_ and preacidification (e.g., to pH 6.5); (2) gelation or curd formation involving the primary enzymatic phase, secondary phase (aggregation of CNs in presence of Ca ions which neutralize the negatively charged residues and form crosslinks between para‐casein micelles) and milk clotting; (3) postgelation treatments that turn the curd into the final consumer product (cheese). As mentioned earlier, the enzymatic hydrolysis of κ‐CN (Figure 1b) is the most critical step during cheese‐making because it is the prerequisite for curd formation. Thus, fully understanding the extent and kinetics of rennet action on κ‐CN is essential. Most results presented in the literature were obtained at gelation point which is one of the parameters commonly used to characterize the rennet coagulation behavior of milk (Lu et al., 2017; Lucey & Fox, 1992; Zhao & Corredig, 2016) as well as to test the rennet activity and its partitioning (Kayihura et al., 2022). Values at this phase are, therefore, very important as they represent the extent of hydrolysis necessary to induce CN aggregation. The values obtained at different times along the enzymatic phase can also reflect the conversion rate.

It is generally assumed that over 85% κ‐CN hydrolysis is necessary to induce the aggregation phase (Dalgleish, 1979; Green et al., 1978) or nearly complete at clotting stage (McMahon et al., 1984; Wilson & Wheelock, 1972). However, as shown in Table 1 and other studies not considered in this review (Table S1), values vary widely due to several factors discussed below. Moreover, considering conditions relevant to cheese‐making such as bovine whole or skim milk with at least 10% solids, pH 6–6.7, a normal clotting time of ~20 min after chymosin addition (Lu et al., 2017), or presence of 0.01%–0.02% CaCl_2_, the experimental data found in the literature provide sufficient evidence to support a degree of κ‐CN hydrolysis <80%. For example, a recent study on fresh milks at pH 6.5 indicates a degree of κ‐CN hydrolysis of 52%–67% after 40 min (Nilsson et al., 2020). Similarly, ~80% para‐κ‐CN release found after 40 min indicates that gelation occurred at a much lower degree of κ‐CN hydrolysis based on the clotting time (6–7 min) shown for unheated reconstituted skim milk (Anema et al., 2007). Eshpari et al. (2015) also found a degree <70% CMP release at gelation point of 21 min. Furthermore, although Lieske et al. (1996) reported 90% GMP release estimated at the time milk clotted, CMP release was 60%, indicating that the total macropeptide release was about 70% considering a GMP proportion of 38%–39% indicated by the same authors. According to He (1990) and van Hooydonk, Boerrigter, and Hagedoorn (1986), the degree of κ‐CN hydrolysis necessary for the onset of aggregation at a standard milk concentration (12% TS) is 60%, also in agreement with Sinaga et al. (2016) for a commercial pasteurized skim milk at pH 6.62.

**FIGURE 2:**
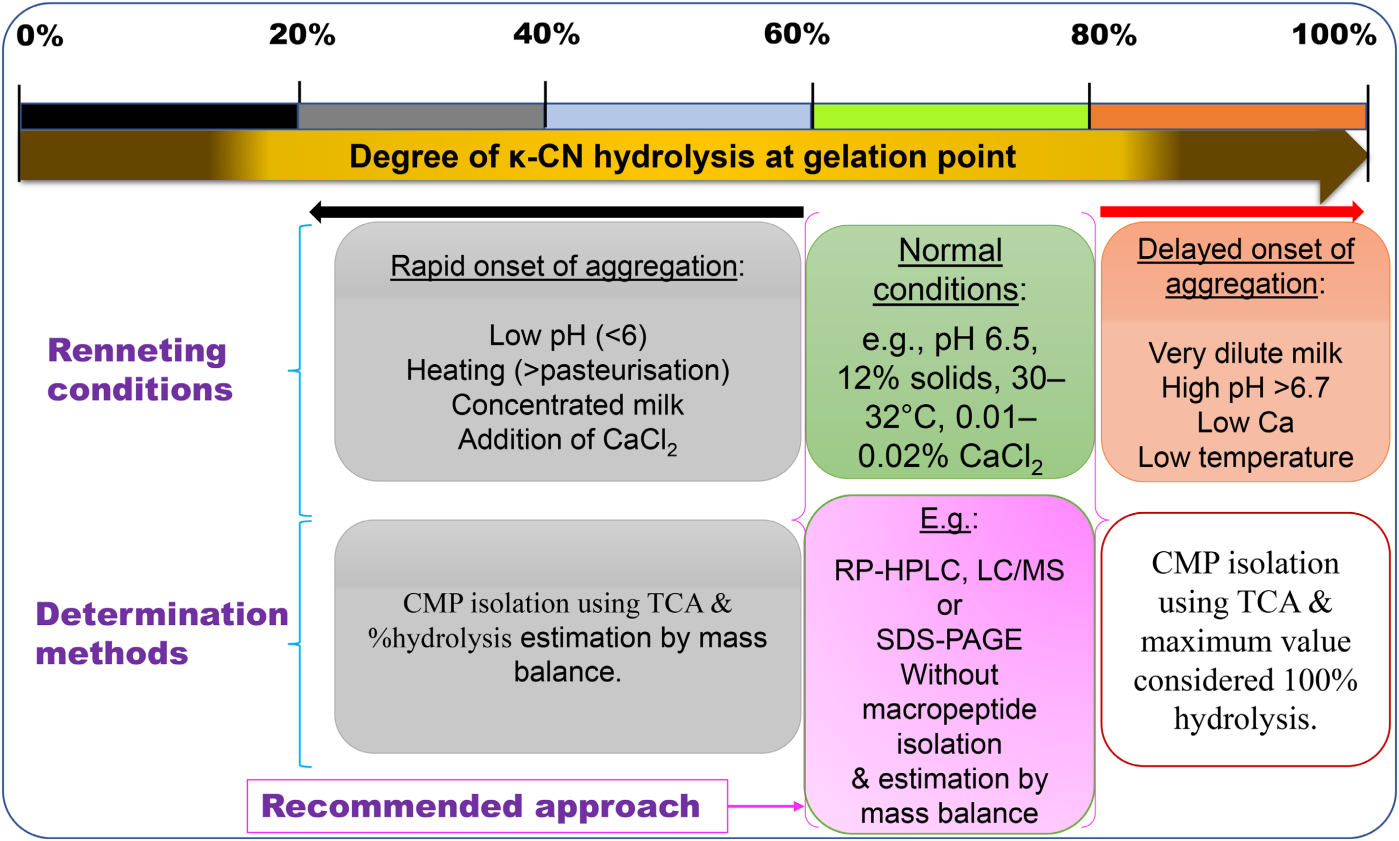
Perspectives on factors affecting the degree of κ‐CN hydrolysis estimated at gelation point.

He (1990) argued that higher degree of κ‐CN hydrolysis quoted in many studies based on a kinetic study by Dalgleish (1979) is only applicable to highly diluted milk. Under very dilute conditions, He (1990) and others (Bringe & Kinsella, 1986a, 1986b; Dalgleish, 1979; Dalgleish et al., 1981; Pierre, 1983) agree on a minimum ~90% hydrolysis at clotting although at a normal renneting temperature of 30°C, Carlson et al. (1987b) also showed a critical conversion of 60% for reconstituted skim milk with 2% solids. In addition, another most cited study is that of Green et al. (1978) in which 86% hydrolysis at the start of rise in viscosity was reported based on an increase in Abs_217_; however, the macropeptide isolated was GMP only. In general, the degree of κ‐CN hydrolysis >80% at gelation point is most likely due to partial estimates of the total amount of the macropeptides released, conditions retarding the aggregation phase allowing longer reaction times, or maximum values considered 100% (Figure 2). For longer reaction times, 100% were reported in several studies (Table 1) but with no evidence that these are actually total values as they were not estimated based on initial κ‐CN content in milk. This is very important because as discussed later, calculations based on an underestimated total value could certainly lead to higher estimates of the degree of κ‐CN hydrolysis than actual values.

## 5 FACTORS AFFECTING THE ESTIMATED DEGREE OF κ‐CN HYDROLYSIS

### 5.1 Milk, coagulant, and additive concentrations and pH

Enzyme‐to‐substrate ratio is a critical factor when studying the kinetics of κ‐CN hydrolysis as the rate was found to increase proportionally with the amount of rennet (Bingham, 1975; Castle & Wheelock, 1972; Sandra et al., 2007; van Hooydonk et al., 1984) and decreases linearly with an increase in CN concentration (Le Feunteun et al., 2012). For a model solution of 1% κ‐CN and rennet applied at a rate of 0.1 μg/mL, about 40% of intact κ‐CN was found after 30 min of reaction whereas a considerable reduction was evident when rennet was increased to 1 μg/mL (Bingham, 1975). On the other hand, at a constant pH of 5.8 and rennet concentration of 0.01 IMCU/mL, a degree of κ‐CN hydrolysis <20% was reported for skim milk concentrated 7× (19.8% CN content) by ultrafiltration (UF) (Karlsson et al., 2007). van Hooydonk et al. (1984) attributed the retarded enzymatic phase for UF‐concentrated milk to low effective diffusion rate of the enzyme. In contrast, it was reported (Sandra et al., 2011; Zhao & Corredig, 2016) that skim milk concentration by UF up to 5× did not affect the enzymatic phase. Furthermore, using the same rennet concentration of 0.00768 IMCU/mL, He (1990) showed that the extent of hydrolysis at gelation point (68%) for reconstituted skim milk with 36% solids was slightly higher than that obtained with a 12% TS sample (60%). Similar results (60%–67%) were obtained with 10% reconstituted skim milk using 0.0036 IMCU/mL (Renan et al., 2007). Unlike disagreements between some studies regarding the degree of κ‐CN hydrolysis at gelation point, the maximum is independent of both milk protein and coagulant concentrations (Castle & Wheelock, 1972; Garnot & Corre, 1980).

Increasing ionic strength of milk by the addition of CaCl_2_ or NaCl may promote or retard both phases of renneting depending on the concentration (Famelart, 1994; Famelart et al., 1999; Klandar et al., 2007). Adding NaCl to cheese milk is most common in Domiati‐style cheeses only, whereas for most other cheeses, CaCl_2_ is often added to milk before renneting in order to increase the aggregation rate of para‐CN micelles, reduce the gelation time, and improve curd firmness and yield (Sandra et al., 2012). The degree of κ‐CN hydrolysis at gelation point decreases with increasing concentration of CaCl_2_ to values ≤ 0.05 M. A reduction estimated at that point was about 3%–10% when 0.6–1.8 mM CaCl_2_ was added to skim milk although the effect was not found when the pH was readjusted to 6.7 (He, 1990; Sandra et al., 2012; van Hooydonk, Hagedoorn, & Boerrigter, 1986). The authors believed that this was because of a rapid aggregation as gelation time decreased linearly with Ca concentration, and a reduction in pH is also clearly one of the reasons as suggested in other studies (He, 1990; van Hooydonk, Hagedoorn, & Boerrigter, 1986; Zhao & Corredig, 2016). With the addition of 10–120 mM CaCl_2_, values could fall to 41%–56% for skim milk with 12%–36% solids (He, 1990), 45% for native phosphocaseinate (Famelart et al., 1999) or 40% for milk protein concentrate (Sandra & Corredig, 2013). In contrast, 0.12 M NaCl showed no effect (Famelart et al., 1999) whereas 0.3 M had a negative impact (Zhao & Corredig, 2016).

The rate of enzymatic hydrolysis appears to increase with both lowering gelation pH (in the range 6.2–6.7) and increasing gelation temperature (2–32°C) (Carlson et al., 1987a) and it was also suggested that slight acidification (to pH 6.3 and 6.5) makes the effect of concentration greater (He, 1990). In contrast, lowering pH in the range below 6 decreased the degree of the macropeptide release found at gelation point of skim milk to 75% (Li & Dalgleish, 2006) or 50% (Karlsson et al., 2007) at pH 5.8, 43% at pH 5.2 (Pierre, 1983), 55% at pH 5.4 (Li & Dalgleish, 2006) and pH 5.6 (van Hooydonk, Boerrigter, & Hagedoorn, 1986), or 11%–26% at pH 5.4–5.3 (Cooper et al., 2010). As indicated in Table 2, the differences in %CMP release shown by different authors at pH 5.4 and 5.8 could be due to different isolation techniques. It is noteworthy that gelation at low pH occurs at a relatively lower degree of hydrolysis because of a synergistic destabilizing action of the two coagulants (acid and rennet) on CN micelles.

### 5.2 Preheating of milk

The impact of heating milk appears to have been studied extensively and was also reviewed (Britten & Giroux, 2022; Kethireddipalli & Hill, 2015) but the effects of renneting conditions and test methods were not comprehensively discussed. It is generally believed that pasteurization or preheating milk up to 90°C/30 min has little impact on the primary phase/κ‐CN hydrolysis (Anema et al., 2007, 2011; Marshall, 1986; Vasbinder et al., 2003). However, Calvo (1995) reported that even 60°C/30 min in the presence of whey proteins inhibited CMP release from micellar CN, and up to 47% reduction was found when 80°C was applied whereas at 85°C/10 min, about 26% reduction was found (Reddy & Kinsella, 1990).

Similarly, 80°C/5 min considerably slowed down the rate of soluble nitrogen and GMP‐carbohydrate groups released during renneting (Beeby & Nitschmann, 1963; Hindle & Wheelock, 1970). In addition, it has been shown that both preheating and preheating followed by homogenization of skim milk reduced the %CMP release by 10%–15% (Sandra & Dalgleish, 2007). A 5%–30% reduction in degree of hydrolysis after 4 h of renneting for milk pre‐heated at 90°C/10 min was also reported depending on the isolation method (Vasbinder et al., 2003). The authors concluded that the reduction was very slight, Ca_3_(PO4)_2_ precipitation has no impact, and whey protein denaturation retards the kinetics of aggregation stage only, which contradicts Calvo (1995) as indicated above. Using half of the rennet concentration used by Vasbinder et al. (2003), Renan et al. (2007) found 10%–17% reduction after 7 h of renneting for reconstituted skim milk heated at 90°C/10 min. It is very clear that the rennet concentrations applied in these two studies were very low based on the normal 20‐min clotting time mentioned earlier. When milk was heated to 100°C for 10 min, the amount of GMP released at the formation of a stiff gel was only 32.7% of the value obtained with unheated milk (Taylor & Woonton, 2009).

Contrary to Vasbinder et al. (2003), several other studies (Calvo, 1995; Lieske, 1997; Reddy & Kinsella, 1990; Wilson & Wheelock, 1972) had suggested that denatured β‐LG and dissociated κ‐CN complexation via hydrophobic interactions and covalent (disulfide) bonds (Anema, 2020; Reddy & Kinsella, 1990) and changes in Ca distribution were the reasons for inhibition of the primary phase due to partial inaccessibility of part of κ‐CN to the enzyme. In addition, van Hooydonk et al. (1987) also concluded that denatured β‐LG/κ‐CN complexation has an adverse effect. Not only as a consequence of heat treatment but also native whey proteins have been confirmed to possess inhibitory properties on κ‐CN hydrolysis (Gamlath et al., 2018). Similar to results shown by Renan et al. (2007), the reduction in the enzymatic rate or CMP release has been shown to be 18% for temperatures ≥90°C but up to 25%–45% have also been found for ultra‐high‐temperature (UHT)‐treated milk (Ferron‐Baumy et al., 1991; Leaver et al., 1995; van Hooydonk et al., 1987). A 25% reduction in CMP release was also found for high‐heat reconstituted milk relative to that of raw milk (Lieske, 1997) and medium‐heat reconstituted milk compared with low‐heat milk (Klandar et al., 2007). For GMP, UHT caused a 40% reduction in the final release compared with raw milk whereas CMP was not impacted (Ferron‐Baumy et al., 1992). For this reason, the authors concluded that complex formation with denatured β‐LG involves only the glycosylated form of κ‐CN. This study also confirms the 17%–18% reduction shown above based on the proportion of GMP indicated (42% of total macropeptide). According to the results reported by Lieske (1997), CMP was the most affected which contradicts Ferron‐Baumy et al. (1992). Based on the isolation techniques, findings of Hindle and Wheelock (1970) also clearly indicate that GMP was less affected by milk sterilization. However, the final amount of total macropeptide and changes in the final amount of GMP‐carbohydrate groups (except D‐galactose, Gal) were lower, indicating that both isoforms decreased.

### 5.3 Methods used to determine the degree of κ‐CN hydrolysis

As shown in Table 2 and other studies not considered in this review (Table S1), measuring the degree of κ‐CN hydrolysis can be achieved using different methods most of which are highly sensitive and provide accurate measurements, for example, reverse‐phase high‐performance liquid chromatography (RP‐HPLC) which is the most common (Table 2) and liquid chromatography coupled with mass spectrometry (LC–MS) (Jensen et al., 2015; Mollé & Léonil, 2005). Other analytical techniques also include cation‐exchange chromatography (Léonil & Mollé, 1991); Kjeldahl (Klandar et al., 2007); sodium dodecyl sulfate/urea polyacrylamide gel electrophoresis (SDS/urea‐PAGE) (Anema et al., 2007; Brinkhuis & Payens, 1985; Chen et al., 2021); and capillary electrophoresis (CE) (Leite Júnior et al., 2019; Nilsson et al., 2020). Nonetheless, the approaches or experimental designs (especially CMP and/or GMP isolation techniques) and calculations found in numerous studies show some drawbacks.

The most common isolation technique involves treatment of renneted samples with 2, 8, or 12% TCA. This precipitates whey proteins and residual CN and also stops the enzymatic reaction (Jensen et al., 2015; Klandar et al., 2007; Sandra et al., 2012). The reaction may also be stopped using pepstatin solution (Brinkhuis & Payens, 1985; Vasbinder et al., 2003). After stirring, the sample–TCA mixture may be incubated for ≥30 min (Klandar et al., 2007; Sandra et al., 2012; Vasbinder et al., 2003) or not incubated (Jensen et al., 2015), and then centrifuged and filtered (0.22 or 0.45 μm). The supernatants (supposedly containing all the macropeptides released from κ‐CN) are collected and used for CMP and/or GMP analysis, for example, using RP‐HPLC or LCMS. As indicated in Table 2, the maximum peak area (for each sample or for fresh milk) is often considered 100% macropeptide release. However, there are experimental evidence indicating that macropeptide isolation influences the amount of the macropeptide obtained and can result in ~30%–35% variation in the degree of hydrolysis estimated (Taylor & Woonton, 2009; van Hooydonk et al., 1987; Vasbinder et al., 2003). For example, although a TCA concentration of 8% was reported as the optimum (van Hooydonk & Olieman, 1982; van Hooydonk et al., 1987), Léonil and Mollé (1991) reported a macropeptide recovery of 30%–75% and van Hooydonk et al. (1984) found a decrease in the amount of CMP recovered of 61%, of that obtained using 2% TCA. Likewise, the results reported by Beeby and Nitschmann (1963), Mackinlay and Wake (1971), and Garnot and Corre (1980) also indicate that 12% TCA isolated only 25%–37.5% of total macropeptide obtained using 2% TCA or at pH 4.7.

It is believed that 6% TCA (+Na_2_SO_4_) and 12% TCA selectively isolate CMP and GMP, respectively, whereas 2% TCA isolates total macropeptide (Boutrou et al., 2008; Lieske, 1997; Lieske et al., 1996; Mackinlay & Wake, 1971; Pierre, 1983; Shin & Jang, 2002; Vasbinder et al., 2003). However, Vasbinder et al. (2003) showed that some CMP A were present in 12% TCA. In addition, the amount of carbohydrates in GMP (N‐acetyl neuraminic acid (NeuAc), D‐Gal and 2‐acetamido‐2‐deoxy‐D‐galactose) estimated using 12% TCA were lower than that found in 2% TCA (Hindle & Wheelock, 1970; Shin & Jang, 2002), an indication that GMP may also be underestimated when 12% TCA is used. Furthermore, although the initial rate of increase in 2% TCA‐soluble NeuAc was slightly greater than that obtained by 10% TCA, the final amount was almost the same whereas differences in the amount of nitrogen released were higher (Wheelock & Knight, 1969). On the other hand, Vreeman et al. (1986) indicated that the optimum concentration of TCA varies depending on the κ‐CN isoform where, for example, 3, 7, and 12% TCA were optimal for κ‐CN BB‐1P, κ‐CN BB‐1P,3NeuAc, and κ‐CN BB‐1,6NeuAc, respectively. However, studies using any amount of TCA are inaccurate according to Thomä et al. (2006), because even 1% TCA showed 10% CMP loss. Léonil and Mollé (1991) and Mollé and Léonil (2005) also agree with Thomä et al. (2006) that due to variations in sensitivities of different isoforms, TCA does not isolate total macropeptide.

All studies above clearly indicate that the isolation method is one of the major factors influencing the degree of κ‐CN hydrolysis reported, but another issue is also how the results are calculated and presented. As shown in Table 2, most studies have presented the results as % of values obtained using unheated/fresh milk or the maximum peak areas for each sample assumed to be 100% (Bansal et al., 2007; Kethireddipalli et al., 2011; Nair & Corredig, 2015; Sandra & Dalgleish, 2007; Sandra et al., 2012; Sinaga et al., 2016; Taylor & Woonton, 2009; Titapiccolo, Alexander, & Corredig, 2010; Titapiccolo, Corredig, & Alexander, 2010). Nonetheless, CMP at a plateau or maximum peak area is not necessarily total macropeptide (100% hydrolysis), especially for reconstituted or heated milk in which complexes containing intact κ‐CN (Kayihura, 2023a, 2023b) may inhibit complete hydrolysis. A good example to explain this is the results presented by Rocha et al. (2021) which indicate that CMP release began to plateau after 30 min but the degree of κ‐CN hydrolysis estimated after 120 min was still <10% because calculations were based on initial total protein in samples. The problem, however, is that Rocha et al. (2021) used 10% TCA which does not recover total macropeptide as discussed above. Another example is a study on pepsin‐induced hydrolysis using RP‐HPLC in which the peak area of para‐κ‐CN produced after 8 h was considered 100% hydrolysis; however, chromatograms indicate the presence of intact κ‐CN especially the B variant (Yang et al., 2022). It becomes clear, therefore, that the measurement procedures and calculation approaches need some improvements.

Since advanced analytical techniques such as SDS/urea‐PAGE, CE, RP‐HPLC, or LC–MS are capable of simultaneously separating individual CNs (including residual κ‐CN), serum proteins and the peptides (para‐κ‐CN & CMP/GMP) in the renneted milk sample, macropeptide isolation seems unnecessary. This is because sample preparation for residual κ‐CN or para‐κ‐CN analysis does not isolate those from other milk components. Instead, aliquots of a whole denaturing buffer‐sample mixture are injected, for example, into HPLC or polyacrylamide gel (Anema et al., 2007; Nilsson et al., 2020; Thomä et al., 2006; Yang et al., 2022). Therefore, the degree of κ‐CN hydrolysis can be estimated based on the amount of residual intact κ‐CN or para‐κ‐CN instead of CMP/GMP as explained below. Moreover, one mole of κ‐CN produces one mole of each of the hydrolysates (para‐κ‐CN and CMP or GMP) as shown in Figure 1b; thus, the best and provable approach to determine the degree of κ‐CN hydrolysis is to do calculations by mass balance:

$${\mathrm{\kappa CN}}_{h}={\mathrm{\kappa CN}}_{I}-{\mathrm{\kappa CN}}_{r}\;\text{Approach}-1$$

and

$$\kappa CN_{h}=\sum_{i=1}^{3}P_{i}\text{Approach}-2$$

where ${\mathrm{\kappa CN}}_{I}$ is the initial κ‐CN content (mg/g of milk), ${\mathrm{\kappa CN}}_{h}$ is the hydrolyzed fraction of κ‐CN (mg/g of milk), ${\mathrm{\kappa CN}}_{r}$ is the residual intact κ‐CN (mg/g of milk), and $P_{i}$ is the total concentration (mg/g of milk) of all the peptides produced (para‐κ‐CN, CMP, and GMP).

Then, the degree of κ‐CN hydrolysis is simply the % ratio of the hydrolyzed fraction of κ‐CN to the initial amount in milk and can be calculated as:

$$100\left(\frac{{\mathrm{\kappa CN}}_{h}}{{\mathrm{\kappa CN}}_{I}}\;\right)=100\;\left(1-\frac{{\mathrm{\kappa CN}}_{r}}{{\mathrm{\kappa CN}}_{I}}\right)$$

Note that the whole renneted sample (without whey separation) should be used to analyze ${\mathrm{\kappa CN}}_{r}$. Also, the rennet action must be stopped at a specific stage/time when the degree of hydrolysis is to be determined. Approach‐1 would give a more direct and the most accurate estimate since ${\mathrm{\kappa CN}}_{I}$ and ${\mathrm{\kappa CN}}_{r}$ can be determined using the same analytical method as mentioned above. The major drawback of approach‐2 as mentioned earlier is that accurate determination of total CMP + GMP released is challenging. As stated above, para‐κ‐CN can also be determined by the same analytical method used for intact κ‐CN (again in the whole renneted sample); therefore, another reliable alternative approach would be based on molar ratios, that is, ${\mathrm{\kappa CN}}_{h}$ can be estimated by multiplying its molecular weight by moles of para‐κ‐CN since one mole of κ‐CN produces one mole of para‐κ‐CN as mentioned above. The degree of κ‐CN hydrolysis can also be expressed as a % ratio of the peak area of para‐κ‐CN at a specific time to the total peak area as shown by Yang et al. (2022, 2023), but complete hydrolysis must be achieved and verified by mass balance calculations, that is, $\kappa CN_{I}=\sum_{i=1}^{3}P_{i}+{\mathrm{\kappa CN}}_{r}$.

To express the degree of κ‐CN hydrolysis in terms of % macropeptide (CMP + GMP) release, development of an accurate approach for determination of total macropeptide (% of κ‐CN) is required. For unheated milk, complete hydrolysis could be achieved by renneting part of the same milk (control) at low temperature (to inhibit micellar aggregation) until no intact κ‐CN is remaining. This should also be verified by mass balance calculations.

## 6 CONCLUSIONS AND FUTURE RECOMMENDATIONS

A full understanding of the extent and kinetics of rennet action on κ‐CN is essential for proper control of the cheese‐making process and determination of κ‐CN's partitioning between cheese and whey. The literature revealed that the degree of κ‐CN hydrolysis estimated at gelation point of bovine milk renneted under conditions relevant to cheese‐making is <80% and varies depending on three major factors: compositional (e.g., enzyme‐to‐substrate ratio), pretreatments (e.g., changing ionic strength and preheating), and test and estimation approaches (e.g., TCA concentration and considering the maximum macropeptide released 100% κ‐CN hydrolysis). The literature also shows that there appears to be little advancement in analytical and estimation approaches since 1950s; thus, the following are recommended for future studies: (1) using advanced analytical techniques (e.g., RP‐HPLC or LC–MS) without macropeptide isolation (i.e., analyzing the residual intact κ‐CN or para‐ κ‐CN in the whole renneted milk instead of isolated CMP and/or GMP), (2) estimating the degree of κ‐CN hydrolysis by performing mass balance calculations (based on initial κ‐CN content in milk), (3) improving an approach to determine total macropeptide release especially in heated and reconstituted milks also remains to be established since there is no clear evidence that complete hydrolysis could be achieved due to κ−/αs_2_‐CNs‐whey protein complexation, and (4) the best method (sample preparation, analytical technique, and procedure) and the actual extent of κ‐CN hydrolysis (%) necessary to induce gelation in various milk systems or renneting conditions (e.g., for specific cheese varieties) will also need to be established.

## CONFLICT OF INTEREST STATEMENT

The author declares no conflict of interest.

## Supporting information


**Table S1.** Other studies that reported the degree of κ‐CN hydrolysis or CMP and/or GMP release (%) but did not meet all the criteria of the review.

## Data Availability

Data sharing is not applicable to this article as no new datasets were created.
