# Supplementary material for: Extent of κ‐casein hydrolysis during renneting of bovine milk: A critical assessment of the analytical and estimation approaches
Source: Food Sci Nutr. 2023 Dec 12;12(3):1399–412. doi: 10.1002/fsn3.3868 (PMC10916671; doi:10.1002/fsn3.3868)
Supplement: Supplementary file 1 — Table S1. Other studies that reported the degree of κ‐CN hydrolysis or CMP and/or GMP release (%) but did not meet all the criteria of the review. [file FSN3-12-1399-s001.docx]

**Table S1. Other studies that reported the degree of κ-CN hydrolysis or CMP &/or GMP release (%) but did not meet all the criteria of the review**

| Degree (%) | Stage or time | Sample type | Amount of rennet | Coagulant type & strength | Reference |
| --- | --- | --- | --- | --- | --- |
| 54 | Final | Whole milk | Not indicated | Chr. Hansen, strength not shown | Wheelock & Knight, 1969 |
| 100 | 20 min | Whole milk | Not indicated | Not indicated | Wilson & Wheelock, 1972 |
| 86 | At the start of rise in viscosity | Skim milk | 0.001 or 0.002% | Not indicated | Green, Hobbs, Morant & Hill, 1978 |
| ~80 or 100, respective of rennet amount | 60 min | κ-CN (1%) | 0.1 or 1mg/mL | Crystalline rennin, 81 units/g | Bingham, 1975 |
| 86-88 | Gelation point of diluted milk at 25°C | 60× diluted skim milk | Soluble or immobilised (IMCU not clear) |  | Dalgleish, 1979 |
| 85 | Start of molecular weight increase | Fresh skim milk | Not clear | Rennet, strength not shown | Dalgleish, Brinkhuis & Payens, 1981 |
| 43 | Gelation point | Skim milk, pH 5.2 | 0.01% | Rennet powder | Pierre, 1983 |
| 92 | Gelation point | Skim milk, pH 6.7 | 0.01% |  |  |
| 100 | Gelation point | Skim milk, pH 7.2 | 0.01% |  |  |
| 7 | Gelation point | κ-CN (0.5 mg/mL in SMUF) | 0.1 SU | Rennet, 10900 SU | Brinkhuis & Payens, 1985 |
| 47.5 | Gelation point | 10× diluted raw skim milk (pH 6.75) with 20 mM CaCl2 | 1.96-9.95×10^-6^ mol/mol κ-CN | Chymosin, no strength shown | Bringe & Kinsella, 1986a |
| 26.8 or <60 | Clotting point (respective of mM CaCl2) | Raw skim milk diluted 10×, pH 6.7 with 60 or 10 mM CaCl2 | Not given | Not indicated | Bringe & Kinsella, 1986b |
| 100 | 60 min | Skim milk, pH 5.6 | 0.01% | Liquid rennet, 10800 Soxhlet Units | van Hooydonk, Boerrigter & Hagedoorn, 1986 pp297 |
| 100 | 100 min | Skim milk, pH 6.7 | 0.01% |  |  |
| 100 | 35 min | Skim milk, pH 6.2 | 0.01% |  |  |
| >80 | Up to 5 h | Skim milk, pH 7.3 | 0.02% |  |  |
| 40 | Up to 5 h | Skim milk, pH 7.5 | 0.02% |  |  |
| 94 | Gelation point (75 min) | Skim milk, pH 6.7 | 0.01% |  |  |
| 86 | Gelation point (18 min) | Skim milk, pH 6.2 | 0.01% |  |  |
| 55 | Gelation point (10 min) | Skim milk, pH 5.6 | 0.01% |  |  |
| <30 | Start of aggregation | Skim milk, pH 5.6 | 0.01% |  |  |
| 95 | Gelation point (30 min) | Skim milk, pH 6.7 | 0.02% | Not indicated | van Hooydonk, Hagedoorn & Boerrigter, 1986 pp 369 |
| 92 | Gelation point (26 min) | Skim milk, with 0.6 mM CaCl2, pH 6.7 | 0.02% |  |  |
| 87 | Gelation point (21 min) | Skim milk, with 1.8 mM CaCl2, pH 6.7 | 0.02% |  |  |
| 89 | Gelation point of unheated milk | heated milk (75°C/30 min) | 0.025% | Standard cheese rennet, strength not indicated | Marshall, 1986 |
| 96 | Gelation point of unheated milk | Heated milk (80°C/30 min) | 0.025% |  |  |
| 100 | 3 hrs | κ-CN solution, pH 6.6 | 0.05% | Chymosin, 6.5×10^6^ Soxhlet units | Vreeman, Visser, Slangen, & Van Riel, 1986 |
| >90 | After 3 hrs | Heated skim milk (≤90°C/10 min) | 2 mL/kg | Liquid rennet, 10800 Soxhlet Units | van Hooydonk, Koster & Boerrigter, 1987pp 3-18 |
| 90 | After 3 hrs | Heated skim milk (120°C/10 min) | 2 mL/kg |  |  |
| >90 | 30 min | Reconstituted skim milk (10%) | Not clear | Single-strength rennet, 100 units/mL | Carlson, Hill & Olson, 1987a |
| 100 | Gelation point | Reconstituted skim milk (2%) at 15°C | Not given | Not indicated | Carlson, Hill & Olson, 1987b |
| 60 | Gelation point | Reconstituted skim milk (2%) at 30°C | Not given |  |  |
| 90-100 | Gelation point & the maximum | Skim milk, pH 6.6 | Not clear | Cheese-making rennet (Hansen's Ltd) & Crystalline chymosin (E.C. 3.4.23.4) | Chaplin & Green, 1980 |
| 90 | Gelation point | Non-fat dry milk, 4% TS | 0.00768 IMCU/mL | Purified rennet (E.C.3.4.23.4, with >95% chymosin), | He, 1990 |
| 90, 100 | Gelation point (<15 min), maximum | Raw skim milk | 0.026% | Rennet (520 mg of chymosin/L) | Ferron-Baumy, Maubois, Garric & Quiblier, 1991 |
| <90, 97 | Gelation point (<20 min), maximum | Pasteurised skim milk (70°C/1 min) | 0.026% |  |  |
| 82 | Maximum (no clotting) | UHT (140°C/1s) skim milk | 0.026% |  |  |
| <90, 93 | Gelation point (<20 min), maximum | UHT (160°C/0.1s) skim milk | 0.026% |  |  |
| <90, 100 | Gelation point (<20 min), maximum | Raw UF (1.8×) retentate | 0.026% |  |  |
| ~85 | Gelation point (>40 min) & the maximum | UHT - UF (1.8×) retentate | 0.026% |  |  |
| 100 | Maximum | Raw skim milk | 0.026% | Rennet (520 mg of chymosin/L) | Ferron-Baumy, Mollé, Garric & Maubois, 1992 |
| 83 | Maximum | UHT (140/10 s) skim milk | 0.026% |  |  |
| 100 | Maximum | Whole milk (CN:fat ratio of 0.7) | 0.02 U/mL | Clonal chymosin, strength not shown | Leaver, Law & Horne, 1995 |
| 50 | Maximum | Whole milk heated (120°C/1 min & 140°C/1 or 5 min) | 0.02 U/mL |  |  |
| 90 (GMP), 60 (CMP) | Gelation point | Skim milk, pH 5.96 | IMCU not given | Pure calf chymosin (P99) | Lieske, Faber & Konrad, 1996 |
| 98 (GMP) | Gelation point | Skim milk, pH 6.67 | IMCU not given |  |  |
| 90 | Gelation point (~20 min) | raw milk | 0.0143% | Pure natural calf chymosin (P99) | Lieske, 1997 |
| 78 | Gelation point (8.3 min) | Native phosphocaseinate (2.5% CN), pH 6.6 | 1 mL/L (IMCU not given) | Not indicated | Famelart, Graet & Raulot, 1999 |
| 78 | Gelation point (18.3 min) | Native phosphocaseinate (2.5% CN), pH 6.6 with NaCl (0.12 M) | 1 mL/L (IMCU not given) |  |  |
| 45 | Gelation point (~11.7 min) | Native phosphocaseinate (2.5% CN), pH 6.6 with CaCl2 (0.12 M) | 1 mL/L (IMCU not given) |  |  |
| 100 | 90 min | Skim milk | 0.02% | Fermentation produced chymosin (Maxiren 180) | Thomä, Krause & Kulozik, 2006 |
| ~80 | Final (40 min) | Reconstituted skim milk | 0.008% | Pure Chymosin (P99) | Anema, Leea & Klostermeyer, 2007 |
| ~80 | Final (40 min) | Reconstituted skim milk heated (90°C, 15 min) | 0.008% |  |  |
| 65-70 | Flocculation | Fresh skim milk & homogenised fresh skim milk (179 MPa) | 0.00345% | Chymostar Double Strength | Sandra & Dalgleish, 2007 |
| 90 | Gelation point (79 or 72 min) | Fresh skim milk & homogenised fresh skim milk (179 MPa) | 0.00345% |  |  |
| 100 | After 20 min | CN suspension (3% protein), pH 6.5 | 0.02% | Naturen, 140 IMCU/mL | Bönisch, Heidebach & Kulozik, 2008 |
| 95 | Onset of radius change (61 min) | Skim milk with CN volume fraction of 0.1, pH 6.7 | 0.007% | Chymostar, Single Strength | Gaygadzhiev, Corredig & Alexander, 2009 |
| 90, 100 | Start of increase in G', gelation point | Skim milk with CN volume fraction of 0.1, pH 6.7 | 0.007% |  |  |
| 100 | ~40 min | Sodium caseinate, 4% | 0.018 IMCU/mL |  | Titapiccolo, Corredig & Alexander, 2010 |
| 80 | Gelation point | Ultra-high pressure treated micellar CN | 0.00833% of CN | Pure chymosin (EC 3.4.23.4) | Heinrich & Kulozik, 2011 |
| 100 | Final (120 min) | Micellar CN (2.6%) | Not indicated | Rennet Stamix 1150, strength not shown | Wang et al., 2014 |
| <80 | Gelation point (4.7 min) | Standardised milk with small fat globules,1.87 μm (3.2% protein, 1.2% fat) | Not indicated | Not indicated | Luo, Wang, Guo & Ren, 2017 |
| <80 | Gelation point (6.7 min) | Standardised milk with large fat globules, 5.65 μm (3.2% protein, 1.2% fat) | Not indicated |  |  |
| <80 | Gelation point (7.8 min) | Skim milk | Not indicated |  |  |
| <80 | Gelation point (6.5 min) | Raw milk | Not indicated |  |  |
| 100 | 60 min (final) | Standardised milk with small fat globules,1.87 μm (3.2% protein, 1.2% fat) | Not indicated |  |  |
| 100 | 60 min (final) | Standardised milk with large fat globules, 5.65 μm (3.2% protein, 1.2% fat) | Not indicated |  |  |
| 100 | 60 min (final) | Skim milk | Not indicated |  |  |
| 100 | 60 min (final) | Raw milk | Not indicated |  |  |
| <80 | Gelation point (8.7 min) | Micellar CN (0.7% protein in simulated milk ultrafiltrate) | 2.235 IMCU/mL | Chymosin (Chr-Max Powder Extra), 2235 IMCU/g | Zhang et al., 2017 |
| 100 | 60 min (final) | Micellar CN (0.7% protein in simulated milk ultrafiltrate) | 2.235 IMCU/mL |  |  |
| 80 | 30 min | κ-CN solution (4 mg/mL) | 0.0525 IMCU/mL | Recombinant chymosin, 2500 IMCU/g | Leite Júnior, Tribst, Ribeiro, Cristianini, 2019 |
| >90 | 60 min | κ-CN solution (4 mg/mL) | 0.0525 IMCU/mL |  |  |
| 96 | Gelation point (36 min) | Pasteurised (72°C/15 s) skim milk with 2.6% CN | 0.01% | Chymo-Plus (EC 3.4.23.4), IMCU not shown | Lauzin, Bérubé, Britten & Pouliot, 2019 |
| 82 | Gelation point (15 min) | Same sample above adjusted to pH 6.5 |  |  |  |
| 93 | Gelation point (23 min) | UF concentrate (6.8% CN) |  |  |  |
| 83 | Gelation point (15 min) | Same sample above adjusted to pH 6.5 |  |  |  |
| 74 | Gelation point (11.5 min) | Reverse osmosis (RO) concentrate (6.4% CN) |  |  |  |
| 90 | Gelation point (20 min) | Same sample above adjusted to pH 6.5 |  |  |  |
| 100 | After 1 h | Skim milk | 0.03 units/mL | chymosin (R4877), 20 units/mg | Chen et al., 2021 |
| 100 | After 1 h | κ-CN solution | 0.03 IMCU/mL |  |  |
|  |  |  |  |  | Total = 38 studies |

Reference:

Bonisch, M.; Heidebach, T.; Kulozik, U. (2008). Influence of transglutaminase protein cross-linking on the rennet coagulation of casein. *Food Hydrocolloids*, *22*, 288–297. <https://doi.org/10.1016/j.foodhyd.2006.11.015>.

Gaygadzhiev, Z.; Corredig, M.; Alexander, M. (2009). The impact of the concentration of casein micelles and whey protein-stabilized fat globules on the rennet-induced gelation of milk. *Colloids and Surfaces B: Biointerfaces, 68, 154–162.* <https://doi.org/10.1016/j.colsurfb.2008.09.026>.

Heinrich, M.; Kulozik, U. (2011). Study of chymosin hydrolysis of casein micelles under ultra high pressure: effect on re-association upon pressure release. *International Dairy Journal*, *21*, 664–669. <https://doi.org/10.1016/j.idairyj.2011.02.003>.

Lauzin, A.; Bérubé, A.; Britten, M.; & Pouliot, Y. (2019). Effect of pH adjustment on the composition and rennet-gelation properties of milk concentrates made from ultrafiltration and reverse osmosis. *Journal of Dairy Science*, *102*, *3939–3946.* <https://doi.org/10.3168/jds.2018-15902>.

Luo, J.; Wang, Y.; Guo, H.; & Ren, F. (2017). Effects of size and stability of native fat globules on the formation of milk gel induced by rennet: fat globules size affects milk gelation. *Journal of Food Science, 82, 670–678*. <https://doi.org/10.1111/1750-3841.13649>.

Wang, F.; Liu, X.; Hu, Y.; Luo, J.; Lv, X.; Guo, H.; Ren, F. (2014). Effect of carrageenan on the formation of rennet-induced casein micelle gels. *Food Hydrocolloids,* *36*, 212–219. <https://doi.org/10.1016/j.foodhyd.2013.10.004>.

Zhang, Y.; Li, Y.; Wang, P.; Tian, Y.; Liang, Q.; & Ren, F. (2017). Rennet-Induced Coagulation Properties of Yak Casein Micelles: A Comparison with Cow Casein Micelles. *Food Research International*, *102*, 25–31. <https://doi.org/10.1016/j.foodres.2017.09.097>.
